# Vocal fold control beyond the species-specific repertoire in an orang-utan

**DOI:** 10.1038/srep30315

**Published:** 2016-07-27

**Authors:** Adriano R. Lameira, Madeleine E. Hardus, Alexander Mielke, Serge A. Wich, Robert W. Shumaker

**Affiliations:** 1 Evolutionary Anthropology Research Group, Department of Anthropology, Durham University, Dawson Building, South Road, Durham, DH1 3LE, UK; 2 Pongo Foundation, Papenhoeflaan 91, 3421XN Oudewater, the Netherlands; 3 Independent researcher, Durham, DH1 5QU, UK; 4 Department of Primatology, Max Planck Institute for Evolutionary Anthropology, Deutscher Platz 6, 04103 Leipzig, Germany; 5 Research Centre in Evolutionary Anthropology, and Palaeoecology, School of Natural Sciences and Psychology, Liverpool John Moores University, Byrom Street, Liverpool L3 3AF, UK; 6 Institute for Biodiversity and Ecosystem Dynamics, University of Amsterdam, Sciencepark 904, Amsterdam 1098, the Netherlands; 7 Indianapolis Zoo, Indianapolis, IN 46222, USA; 8 Krasnow Institute for Advanced Studies, George Mason University, Fairfax, VA 22030, USA; 9 Anthropology Department, Indiana University, 107 S. Indiana Ave., Bloomington, IN 47405, USA

## Abstract

Vocal fold control was critical to the evolution of spoken language, much as it today allows us to learn vowel systems. It has, however, never been demonstrated directly in a non-human primate, leading to the suggestion that it evolved in the human lineage after divergence from great apes. Here, we provide the first evidence for real-time, dynamic and interactive vocal fold control in a great ape during an imitation “do-as-I-do” game with a human demonstrator. Notably, the orang-utan subject skilfully produced “wookies” – an idiosyncratic vocalization exhibiting a unique spectral profile among the orang-utan vocal repertoire. The subject instantaneously matched human-produced wookies as they were randomly modulated in pitch, adjusting his voice frequency up or down when the human demonstrator did so, readily generating distinct low vs. high frequency sub-variants. These sub-variants were significantly different from spontaneous ones (not produced in matching trials). Results indicate a latent capacity for vocal fold exercise in a great ape (*i*) in real-time, (*ii*) up and down the frequency spectrum, (*iii*) across a register range beyond the species-repertoire and, (*iv*) in a co-operative turn-taking social setup. Such ancestral capacity likely provided the neuro-behavioural basis of the more fine-tuned vocal fold control that is a human hallmark.

Spoken languages are learned anew with every human generation. Great apes, however, our closest relatives, are traditionally thought to be incapable of vocal learning^1^,^2^,^3^,^4^ – the capacity to expand their vocal repertoire with new calls learned from others^5^. This apparent paradox has led to the suggestion that human vocal capacities have no imitative precursor in nonhuman species^6^. The evolution of speech – the predominant means of expression of human language^5^ – is hence currently hotly debated, as evidence seemingly challenges the importance of shared ancestry for the emergence of speech within the primate lineage, even though shared ancestry represents one of the founding pillars of Darwin’s theory of natural selection^6^.

Historical “great ape language projects” have trained captive individuals in the attempt to teach them new word-like utterances^7^,^8^. Results were, however, virtually null^1^,^6^. One major limitation of these landmark studies was the fact that detailed descriptions of the great ape vocal repertoire were, for the most part, unavailable at that time. Importantly, scientists had no verifiable catalogue or database to compare and gauge exhibited vocal flexibility. Ultimately, great apes’ vocal skills were directly compared with humans’, rather than objectively against their own natural vocal preferences, predispositions, and limitations.

This critical drawback has been addressed recently: new databases on the natural vocal behaviour of great apes have allowed recognizing vocal learning of new voiceless consonant-like calls^3^,^9^, notably requiring supralaryngeal control of the vocal tract. A modern-day and informed approach to great ape vocal repertoire could, therefore, also clarify whether (besides supralaryngeal control) vocal learning can also involve vocal fold control. This capacity would permit volitional voice modulation^5^, enabling individuals to expand their repertoire with new voiced vowel-like calls. Together with consonants, vowels represented the building blocks for spoken language. Being able to socially learn new voiceless *and* voiced calls would have, thus, effectively set the evolution of an ancestral hominid articulatory system on a course towards a vocal system fundamentally similar to modern speech. The evolutionary implications of the presence of vocal fold control (or volitional voice modulation^5^) in great apes warrants, therefore, revisiting the “unsuccessful” protocols of previous historical studies under a new lens.

Thus far, great apes have been shown to exercise vocal fold control in some degree in “species-specific” voiced calls (or “vocalizations”), i.e. that are typically produced by the species^10^,^11^,^12^,^13^,^14^. Other studies have shown that a number of individual-specific and population-specific voiced calls in great apes do not conform to genetic and ecological divergence^9^,^15^,^16^, suggesting that vocal fold control may play indeed an active role in shaping the composition of the voiced repertoire of great apes. Together, these data confirm that it is imperative for our understanding on the evolution of spoken language to assess the extent to which human vocal fold skills elaborated upon those present in great apes^17^,^18^.

Here, we report a novel orang-utan vocalization, coined “wookie”, idiosyncratic to the vocal repertoire of an adolescent captive male – named Rocky. Our working hypothesis posed that the study subject produced wookies through volitional control over the vocal folds. If this hypothesis was in fact correct, two major predictions followed. First, vocal fold activity and acoustic profile of the wookie should be clearly different from those of other orang-utan calls. Second, the study subject should be able to adapt vocal fold action in response to random stimuli under rigorous controlled experimental settings (e.g. to rule out arousal-based mechanisms). The calls produced in this fashion should be perceptually distinct according with their respective stimuli.

To test the first prediction and verify the novelty of wookies, we evaluated wookies’ acoustic profile in light of the known orang-utan call repertoire. Specifically, we measured and assessed parameters describing vocal fold activity and supralaryngeal manoeuvring between wookies and its most similar call-type in the orang-utan repertoire. To test the second prediction, we brought the subject’s putative vocal fold control under scrutiny by presenting him with a imitative “do-as-I-do” game paradigm^19^,^20^. Under this paradigm, a human demonstrator produced wookie-approximations with varying acoustic features as an implicit request towards the subject to produce vocalizations of matching features. Subject’s vocal responses were recorded and compared with the human models and between themselves. Our results show that a nonhuman great ape can achieve levels of volitional voice control qualitatively comparable to those manifested in humans; notably, real-time, dynamic and interactive vocal fold control beyond the species-specific vocal repertoire.

## Methods

### Orang-utan wookies and the species-specific repertoire

#### Data Collection

To test the first prediction of this study and verify the idiosyncrasy of wookies and their novelty among the known orang-utan repertoire, we recorded spontaneous wookies from Rocky (studbook ID: 3331) during interactions with the human experimenter (MEH) between April and May 2012 at the Indianapolis Zoo, where he is currently housed. We used a ZOOM H4Next Handy recorder via the inbuilt mic standing on a miniature tripod at approximately ~0.5 m distance from the subject. Recordings were collected at a sampling rate of 24 bit/48,000 kHz and saved in wav format. These settings obtained high quality audio recordings and are standard for the collection of orang-utan call behaviour in captivity and the wild. The original version of wookies has been produced by Rocky for at least the last 6.5 years. It was apparent when the experimenters first met Rocky when he was 3.5 years old. It is unclear how he originally learned the vocalization and no recordings are available from earlier years. Wookies are produced by the subject to gather attention from caretakers^16^,^21^. Recordings from the known orang-utan call repertoire available from previous work^22^ were used in order to draw a comparison with wookies.

#### Data analyses

**Figure 1:**
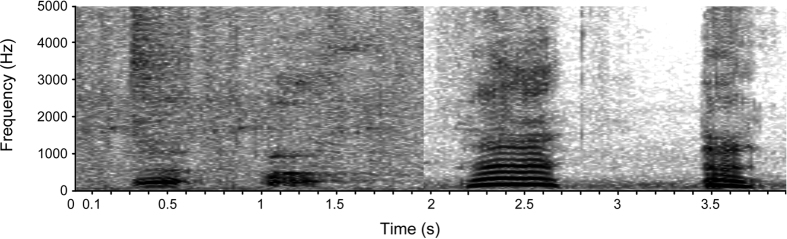
Spectrographic representation of two orang-utan grumphs followed by two wookies.

In order to verify the novelty of wookies in relation to the remaining orang-utan call repertoire, we assessed the largest database ever assembled of orang-utan calls^22^, currently spanning more than 12,000 observation hours across 9 wild and 6 captive populations, and comprising more than 120 individuals. We compared wookies produced spontaneously (i.e. not given in response to human wookie-versions) with the spectrally most similar vocalization known to be produced by orang-utans – the grumph^22^. Grumphs were the only vocalizations presently described in the orang-utan repertoire to exhibit a complete overlap in frequency range with wookies (grumphs: 86–1723 Hz, wookies: 99.6–1418 Hz). Both calls were the only orang-utan vocalizations to fall below 100 Hz and simultaneously reach above 350 Hz^22^ (Fig. 1). Wookies were produced with ingressive air-flow, whereas grumphs were presumably produced with egressive air-flow (as various other orang-utan calls)^22^. Nevertheless, we decided to conduct a comprehensive acoustic comparison in order to verify, with confidence, wookies’ idiosyncrasy and prevent claims of novelty strictly based on one immeasurable articulatory feature (i.e. air-flow direction). For this comparative analysis, grumphs were sampled from wild adolescent males of similar age as Rocky in order to control for the largest number of potentially confounding factors as possible; primarily, sex and body size variation. In order to control for potential geographic variation in grumph acoustics, all wild adolescent males were sampled from the same population (i.e. Ketambe Forest, Aceh, Sumatra, Indonesia).

To acoustically compare wookies with orang-utan grumphs, acoustic measures were conducted with Praat, using “voice report” standard settings, except for voicing threshold in the pitch settings, which was set to 0.15. Seven acoustic parameters describing vocal fold oscillation were measured: duration, median pitch, mean pitch, pitch standard deviation, minimum pitch, maximum pitch and pitch amplitude. Complementary, three acoustic parameters describing supralaryngeal action were measured: first, second and third formant. Because these parameters directly express the position of the tongue and jaw during vocal production, they were used to assess whether wookies also involved different oral manoeuvres, besides different oscillation patterns at the vocal folds.

Statistical analyses were conducted using nonparametric tests with IBM SPSS Statistics 21 (SPSS, Inc.). To compare the differences between wookies and grumphs, one would typically use a Mann-Whitey U test for each parameter. However, because different individuals contributed with several calls to our dataset, this condition violated the assumption of data independence for conducting Mann-Whitney U tests. As such, we opted to conduct Kruskal Wallis tests between individuals for each parameter, while correcting for multiple testing using Bonferroni correction. We expected that Kruskal Wallis test results would show the following. For each parameter, our study subject should be different from all other individuals, while all other individuals should not be different between themselves, since wookies only derived from our study subject whereas grumphs derived from all the remaining individuals. For these analyses, we included our subject and the other adolescent males for whom a sample size larger than one was available (i.e. 2 individuals with 24 and 12 calls). This operation resulted in the exclusion of three adolescent males for which one grumph recording was available.

### Orang-utan vocal fold action in match trials

#### Data Collection

To test the second prediction of this study, experimental testing was conducted with Rocky during April and May 2012 at the Indianapolis Zoo. The zoo’s committee provided ethical approval and permission to conduct research, and the methods were carried out in accordance with the approved guidelines. “Do-as-I-do” paradigm was selected for match trials because this paradigm has been successfully used previously to invoke voluntary call responses in captive orang-utans^19^,^20^. Human demonstrator used protective gloves and a facial mask at all times and interacted with Rocky always through enclosed mesh. Rocky was rewarded during trial sessions with customary food snacks (i.e. raisins and dried plums) or drinks, prepared and provided by full-time orang-utan caretakers at the zoo. Caretakers assured the items used differed in no noticeable way in terms of the subject’s food preferences and food rewards did not vary within trial sessions.

Under the “do-as-I-do” test paradigm, the human demonstrator presented Rocky with random sequences (Runs test, Z = −4.751, p < 0.001) of human wookie-versions varying in frequency (Hz) – low vs. high wookies. 513 trials were presented (272 low, 241 high), divided through 13 sessions (~49 trials/session, ~472 seconds/session) over the course of 5 days. The subject typically responded to the model signal within approximately 500 ms.

Trial sessions were recorded at ~0.5 m distance from the subject with a ZOOM H4Next Handy recorder via the inbuilt mic standing on a miniature tripod. Recordings were collected at a sampling rate of 24 bit/48,000 kHz and saved in wav format. These settings obtained high quality audio recordings. Rocky only joined trial sessions voluntarily and never refused to participate. Rocky was never food deprived during trials sessions and trial sessions never interfered with normal feeding times or working schedule at the orang-utan enclosure so as to prevent imposing any stress. Rocky was tested when he and his cohort (four other orang-utans) were housed in their individual quarters.

During trial sessions, only the first reply immediately after the human model was considered for analyses, unless the human demonstrator verbally instructed (repeating the call model or saying the name of the variant to be matched, “low” or “high”) the focal to repeat, in which case we considered the call produced after the last instruction provided by the human demonstrator, or the last call produced by the focal before the human demonstrator verbally closed the bout (e.g. by saying “yes” or “very good”). We did not consider calls when overlap between human model and orang-utan match reply did not allow suitable extraction of acoustic parameters from both calls (i.e. focal was too quick to reply).

We intentionally selected a human demonstrator with no previous voice training or music experience. Because our main aim was fundamentally evolutionary, we deliberately avoided using a demonstrator with vocal skills well beyond those potentially present in a human ancestor. We mandated model calls to be as “raw” and naturally sounding as much as possible. No *a priori* guidelines were given to the human demonstrator before match trials and no acoustic treatment was given to her utterances. Moreover, we purposefully did not obstruct the human demonstrator from deploying her natural behaviour during the interaction (e.g. occasional approximation to the subject, occasional arm movement). Crucially, this decision allowed the demonstrator to keep the subject engaged and cooperative during the tests. Nevertheless, we were adamant about providing no training sessions, opportunities or time to the subject before the match trials, and the subject was presented a human demonstrator with whom he was not familiar. These factors confidently assured that our subject did not develop conditioned responses.

#### Data analyses

In order to compare the acoustic profile and general vocal fold oscillation between human- and orang-utan-produced wookies, we selected and analyzed call maximum frequency (Hz). This parameter was also used to compare the subject’s wookie sub-variants between each other (spontaneous, high and low). Maximum frequency is the frequency at which maximum energy (dB) occurs within a call. For this reason, maximum frequency contributes disproportionally to pitch and, in the case of wookies, it represented one of the best proxies available for pitch (Spearman test between maximum frequency and mean pitch, r = 0.341, N_spontaneous wookies_ = 124, p > 0.001). Moreover, maximum frequency was equal to the fundamental frequency (F_0_) 93.4% of 500 measured cases. Therefore, maximum frequency provided one of the most reliable measures of the oscillation rate of the vocal folds and its perception. In order assess the subject’s level of accuracy during the task, we also conducted the same test but analysing low and high wookies separately.

Besides maximum frequency, we measured duration and maximum power (dB) within each call. Because all recordings were conducted at a constant distance from the study subject, maximum power could be used as a proxy of glottal air pressure during call production. This measure allowed us, thus, to monitor the contribution of abdominal action (generating air current within the vocal tract) during the production of wookies exhibiting different maximum frequencies.

Maximum frequency, duration and maximum power were extracted from recordings using Raven Pro software package (version 1.5, Ithaca, NY: The Cornell Lab of Ornithology) and Hann type spectrogram grip spacing at 2.93 Hz. The use of other important parameters characterizing vocal fold oscillation (e.g. harmonics-to-noise ratio) was hampered because these parameters are particularly susceptible to recording settings^20^.

Nonparametric statistical analyses were conducted using IBM SPSS Statistics 21 (SPSS, Inc.). Spearman binomial correlation test was used to assess a potential effect of human model calls on the responses produced by the study subject. Wilcoxon signed ranks test was used to identify potential differences between wookie subvariants produced by the study subject. Discriminant function analyses were used to assess whether wookie subvariants produced by the study subject could be distinguished perceptually. Discriminant function analyses were conducted both by setting prior probabilities (i.e. chance probability of correct assignment) equal between all groups and by computing prior probabilities based on group size. Because our data set for these analyses derived from the same individual, this did not require conducting a permuted discriminant function analysis. A permuted analysis would have otherwise allowed controlling for a possible confounding variable. For instance, if several individuals had contributed wookie subvariants, the permuted analysis would have allowed controlling for individual variation while assessing the capacity to correctly distinguish wookie subvariants.

Because receivers sense acoustic signals holistically instead of attending to one or few acoustic parameters separately^23^, we tested whether low and high wookies produced by Rocky were overall perceptually distinct from each other by using automated classification algorithms, combined with artificial neural networks (ANN) and mel frequency cepstral coefficients (MFCC)^24^, a classification method that scans and analyses signals based on their general acoustic profile. These analyses allowed assessing the differences between wookie sub-variants while taking in consideration their complete acoustic profile simultaneously, other than one acoustic parameter at a time. For both feature extraction and network analyses, Matlab R2012b (The MathWorks, Inc., Natick, MS, U.S.A.) was used. The MFCCs in this study were computed using the ‘melcepst’-routine available in the toolbox Voicebox. We optimized both MFCC and ANN according to published guidelines^24^. To acquire a MFCC, each call was sliced into seven frames using a Hamming window, two-thirds frame overlap and 16 mel-spaced filters^24^. We used 10 hidden layer neurons and 100 iterations to obtain an optimal ANN^24^. To increase the reliability of the results, every call was tested against seven neural networks, and the condition proposed by the majority of the networks was considered final^24^. Calls were tested using a leave-one-out procedure^24^.

Lastly, we conducted Spearman binomial correlation tests between maximum frequency, duration and maximum power of the subject’s wookies in order to investigate general production dynamics. With these analyses, we were particularly interested in examining to what extent low and high wookies could have been produced strictly by means of changes in glottal air pressure generated by abdominal control (other than by vocal fold control).

## Results

### Orang-utan wookies and the species-specific repertoire

**Figure 2:**
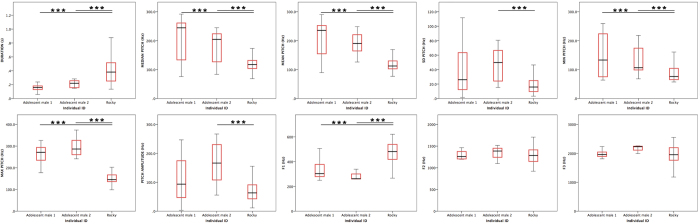
Boxplot per acoustic parameter of Rocky (producing wookies) and other adolescent males (producing grumphs) (middle line represents the median, the box represents the interquartile range (IQ) containing the middle 50% of the data, and the whiskers represent 1.5 times the IQ).

A number of acoustic parameters was measured characterizing the oscillation pattern of the vocal folds with high accuracy. Significant differences were detected within our sample comprised by our study subject (n_wookies_ = 124) and other adolescent males (n_grumphs_ = 36, n_individuals_ = 2, n_grumphs/ind_ = 24, 12) with regards to duration (Kruskal Wallis test, df = 2, X^2^ = 62.080, p < 0.001), median pitch (X^2^ = 29.404, p < 0.001), mean pitch (X^2^ = 56.899, p < 0.001), pitch standard deviation (X^2^ = 20.592, p < 0.001), minimum pitch (X^2^ = 26.508, p < 0.001), maximum pitch (X^2^ = 62.201, p < 0.001), and pitch amplitude (X^2^ = 20.540, p < 0.001). Post hoc pairwise comparisons between individuals revealed that, for all parameters, our study subject was (with the exception of two out of 14 pairwise comparisons) always significantly different from the remaining individuals (duration: p < 0.001 and p < 0.001; median pitch: p < 0.001 and p = 0.002; mean pitch: p < 0.001 and p < 0.001; pitch standard deviation: p < 0.001 and p = 0.054; minimum pitch: p < 0.001 and p = 0.004; maximum pitch: p < 0.001 and p < 0.001; pitch amplitude: p < 0.001 and p = 0.133). At the same time, the remaining individuals showed always no significant differences between each other (duration: p = 0.539; median pitch = 1.000; mean pitch: 1.000; pitch standard deviation: 0.124; minimum pitch: p = 1.000; maximum pitch: p = 0.884; pitch amplitude: p = 0.051). Overall, wookies were significantly longer and exhibited lower pitch values than grumphs (Fig. 2 and Table S1 in Supplementary material).

In addition, we compared in the same way the first, second, and third formant (F1, F2, F3) between our subject and other adolescent males to assess differences in supralaryngeal maneuvering during vocal production. Significant differences within our sample of individuals were found for F1 (Kruskal Wallis test, df = 2, X^2^ = 11.964, p < 0.001), but neither for F2 nor F3 (X^2^ = 0.470, p = 0.791; X^2^ = 2.307, p = 0.316, respectively). Post hoc pairwise comparisons between individuals revealed that our study subject was significantly different from the remaining individuals for F1 (p = 0.037 and p = 0.019), but the remaining individuals were not different between each other (p = 1.000). Overall, tongue body (F2) and tip (F3) positioning was relatively similar between the two calls types but wookies (presenting a higher F1) involved a wider opening of the mouth during call production than that required for grumph production^25^.

These analyses encompassed multiple testing. Correction of significance level was therefore required. Even though Bonferroni correction represents an over-conservative method (0.05/10 = 0.005)^26^, this adjustment did not affect our results on vocal focal action, since all our tests yielding significant differences provided p values smaller than 0.001. The only significant difference dissolved by Bonferroni correction concerned F1 between our subject and the remaining adolescent males. Essentially, this result indicates that differences in vocal fold action provided the most reliable and consistent way of distinguishing wookies versus grumphs, whereas differences in supralaryngeal action were less secure.

### Orang-utan vocal fold action in match trials

**Figure 3:**
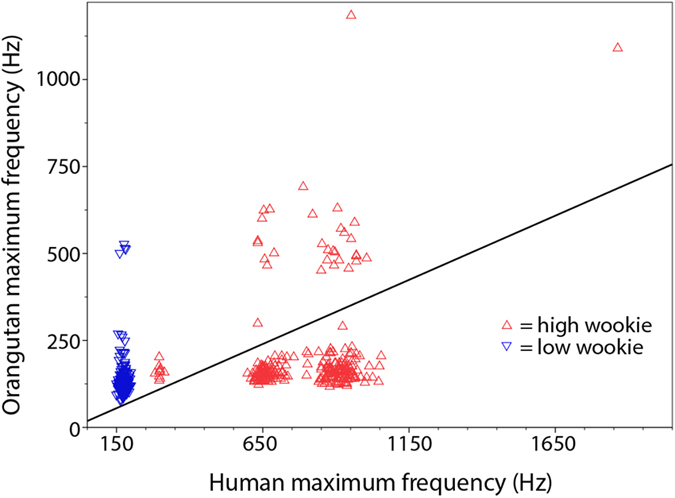
Maximum frequency of human wookie demonstrations against maximum frequency of Rocky’s match wookies (linear fit line with intercept suppressed).

Maximum call frequency (Hz) of human-wookies and orang-utan-wookies showed a significant positive correlation (Spearman, r = 0.574, N = 513, p < 0.001) (Fig. 3). When testing for low and high wookies separately, a significant correlation between human-wookies and orang-utan-wookies was also reached for high wookies (Spearman, r = 0.141, N = 241, p = 0.029).

**Figure 4:**
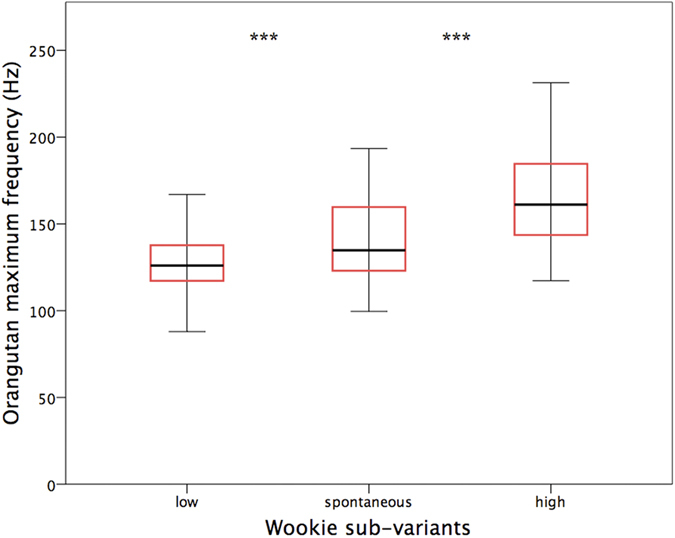
Boxplot of the maximum frequency of low, spontaneous, and high wookie by Rocky (middle line represents the median, the box represents the interquartile range (IQ) containing the middle 50% of the data, and the whiskers represent 1.5 times the IQ).

Maximum frequency differences between low and high wookies produced by Rocky significantly differed from each other (Wilcoxon Signed Ranks test, Z = −10.409, p < 0.001), with low and high wookies exhibiting a median frequency of 126 Hz and 161.1 Hz, respectively, a difference nearly equivalent to a four-note interval on a standard musical octave (B–E) (Fig. 4, Table S2 in Supplementary material). Low and high frequency wookies produced by the subject also significantly differed in maximum frequency from spontaneous wookies (n = 124) (low vs. spontaneous wookies: Wilcoxon Signed Ranks test, Z = −4.405, p < 0.001; high vs. spontaneous wookies: Z = −3.101, p = 0.002), with spontaneous wookies exhibiting an intermediate median frequency of 134.8 Hz (Fig. 4, Table S2 in Supplementary material). Bonferroni correction of our significance value (0.05:3 = 0.0167) did not affect our results.

**Figure 5:**
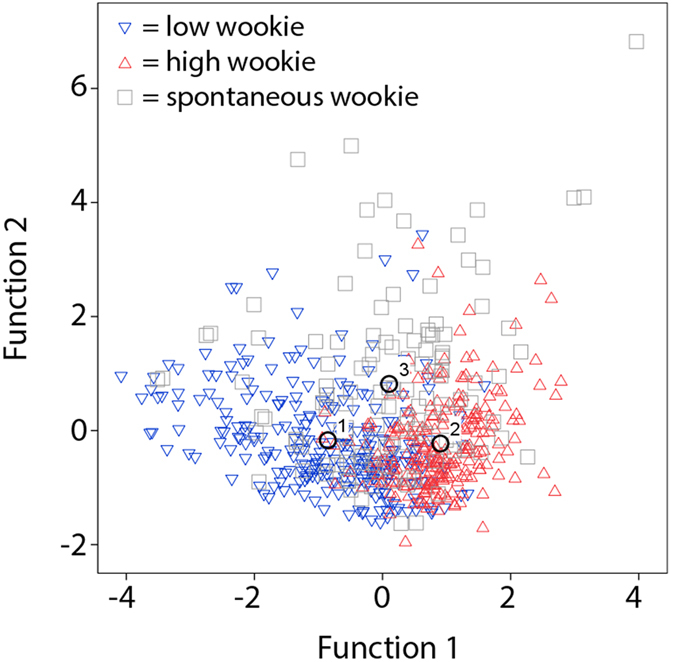
Graphic representation of first and second canonical discriminant functions, displaying distribution and group centroids of Rocky’s low frequency (1), high frequency (2), and spontaneous wookies (3).

Discriminant function analysis, based on maximal frequency alone, attained 50.1% of corrected assignments between low, high, and spontaneous wookies (49.6% using leave-one-out procedure), performing significantly above chance (Wilks’ Lambda Chi-square, X^2^ = 47.128, df = 2, p < 0.001; Binomial test, chance probability = 0.333, p < 0.001). Correct assignments decreased slightly to 48.0% (48.0% using leave-one-out procedure), but remained well above chance, when computing chance levels according to group size (low wookies: 42.6%; high: 38.0%; spontaneous: 19.4%). Percentage of correct assignments to the three sub-variants increased to 69.5% (69.3% using leave-one-out procedure) when supplementing duration and maximum power to the analyses (Fig. 5). In these conditions, maximum frequency (together with maximum power) held the largest absolute correlation with the first discriminant function, which explained 79.4% of the total observed variation. Percentage of correct assignments increased to 72.5% (72.1% using leave-one-out procedure) when computing chance levels according to group size.

These results were corroborated when ascribing the classification of low and high wookies to an automated process scanning the vocalizations’ general acoustic profile. The mean (25%; 75% percentiles) percentage of correct assignments per session was 87.82% (84.82%; 95.12%). Altogether, these results confirmed that low and high wookies were perceptually distinct, and thus, that they could potentially encode biologically pertinent differences.

**Figure 6:**
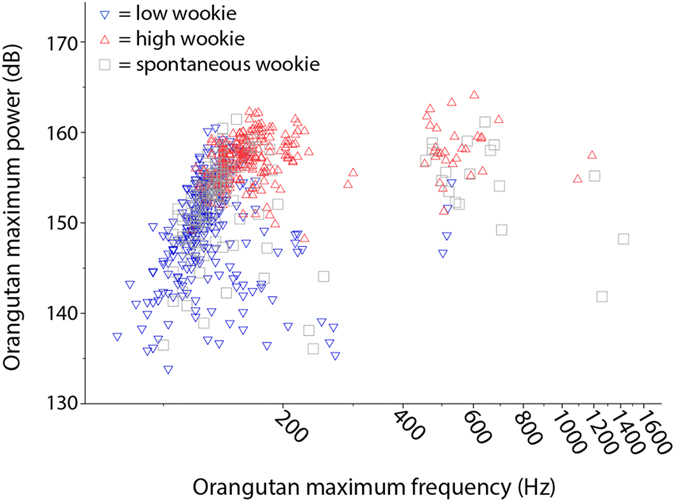
Phonetogram displaying Rocky’s wookies according to maximum frequency and maximum power.

Maximum frequency, duration, and maximum power of Rocky’s wookies showed significant positive correlations (Spearman, n = 639, maximum frequency × duration: r = 0.116, p = 0.003; maximum frequency × maximum power: r = 0.134, p = 0.001). Bonferroni correction of our significance value (0.05:3 = 0.025) did not affect these results. Graphical examination of Rocky’s phonetogram (Fig. 6) showed that at any given sound pressure level Rocky was capable of generating a frequency range wider than 100 Hz. This effect was particularly visible in high frequency wookies, with Rocky producing most of the calls around 160 dB but spanning well above 200 Hz. At the same time, Rocky was able to produce any specific frequency tone across a range of more than 20 dB.

## Discussion

### Orang-utan wookies and the species-specific repertoire

Our results validated our first prediction, showing that wookies represent an acoustically distinct voiced call within the orang-utan call repertoire. Wookies exhibit features of air-flow, vocal fold action and jaw position unique to Rocky and described here for the first time in the *Pongo* genus. These results confirm the capacity of orang-utans to learn and acquire new calls into their individual repertoires, both in the form of voiceless consonant-like calls^3^,^4^,^9^,^15^,^20^ and voiced vowel-like calls^9^,^15^.

Because our analyses focused on an idiosyncratic vocalization, there were inevitable limitations in our statistical analyses. However, after conducting procedures that contemplated the potential of confounding effects, results were always highly significant. Together with the observation that wookies and their closest counterpart in the known orang-utan repertoire exhibit opposite air-flow directions, our analyses allowed determining with confidence that wookies are a novel vocalization based on parameters describing vocal fold oscillation.

Despite an N of 1, our study allows revaluating current assumptions on great ape vocal capacities as well as reformulating some of the basic premises of a general theory of spoken language evolution. By demonstrating vocal learning beyond the species-specific repertoire in a great ape, our results unveil a fundamental parallel with human spoken languages. Namely, the two vocal systems, separated by approximately 10 mya^27^, can be assumed homologous regarding open-endedness and the voiced/voiceless nature of their two building blocks.

### Orang-utan vocal fold action in match trials

Our results validated our second prediction, indicating that the subject modulated vocal fold oscillation according to the model-calls provided by the human demonstrator under controlled settings. The subject adjusted his voice frequency up or down when the human model did so. For this, the subject produced significantly different vocal sub-variants that stood in average outside his normal spectrum of wookie vocalizations. Human demonstrations, thus, effectively guided the subject’s vocal output. Moreover, results suggest that the subject attended, was sensitive to and coordinated his vocal responses according to the spectral dispersion of sub-variants beyond the low/high dichotomy and down to a scale of tens of Hz. Manual and automated procedures demonstrated that his low vs. high wookies exhibited clear perceptible differences, allowing discerning the two with high accuracy.

Correlation between wookies’ acoustic parameters produced by the subject indicated that high frequency wookies were simultaneously louder and longer. That is, high wookies were partly underlined by higher airflow pressure exciting the vocal folds. Accordingly, the production of wookie sub-variants by our subject resulted from the synchronized exercise of the vocal folds and the abdominal musculature generating glottal airflow (e.g. diaphragm). The action of abdominal muscles may have partially alleviated the degree of vocal fold control required to obtain the observed dynamic production across frequencies during match-trials. This positive acoustic interdependence between frequency and glottal air pressure also characterizes, however, overall human vocal production, including people with musical training^28^, and is a phenomenon predicted to be common among animal vocal communication systems. Nevertheless, different wookies produced by Rocky with equal frequencies exhibited wide differences in acoustic power, and vice versa. These observations would have been theoretically impossible if Rocky had not exercised some degree of direct control over vocal fold oscillation, and instead had only resorted to abdominal action to produce modulations at the level of vocal fold oscillation. The subject’s phonetogram attests that vocal fold control was effective and moderately autonomous from abdominal control.

Our match trials were conducted in constant settings in one-to-one interactions between the subject and the human demonstrator. Food rewards were part of the subject’s daily diet and were always kept constant within sessions. Accordingly, we can ascertain that the subject’s performance and vocal output was not affected by the influence of other orang-utans, physical surroundings or food-driven arousal. Thus, the different wookie sub-variants produced by the subject were unrelated to specific changes in context and can be considered to have conveyed no change in function or informational content.

Any possible biasing effects deriving from the natural behaviour of the human demonstrator can also be excluded in light of our results. For example, the demonstrator occasionally approached the subject and moved her arm during low and high vocal models, respectively. The subject could have hypothetically used these supplementary visual cues to know which response was “correct” (instead of directly mimicking the demonstrator’s voice modulation), or these cues could have somehow affected the subject’s arousal in a coherent way with correct responses (“clever Hans effect”). Such interpretations can, however, be dismissed at least for three reasons. First, the subject neither necessarily gazed directly at the human demonstrator to produce a correct response, nor did human supplementary cues ensured subject’s correct responses (see supplementary video). Second, the subject never raised his arm in response to the similar movement by the demonstrator. Thus, he attended to human *acoustic* signals, not other cues. Third, in case the subject’s arousal had been affected, one would expect an increase in subject’s arousal when interacting with a human. However, subject’s low calls were lower than his spontaneous calls. Overall, visual cues or arousal offer no parsimonious explanation for our results.

### Implications for spoken language evolution

Our findings imply the functional presence of direct pathways between the primary motor cortex and the nucleus ambiguous (site of the laryngeal motor-neurons in medulla oblongata) in the ape brain, as observed in an chimpanzee by Kuypers^29^, allowing some degree of vocal fold control autonomous from context and individual’s affective state. Specifically, our analyses indicate that vocal fold control pathways and respective firing in the ape brain integrate with pathways innervating other musculatures engaged in vocal production (namely, abdominal muscles). Several motor maneuvers are brought together synergistically to generate a particular acoustic output.

Contrarily to the notion that spoken language emerged abruptly sometime along the genus *Homo*^30^, our findings amplify the spoken language evolution timeline at least five-fold (assuming speech evolution onset in *Homo* paleodemes, from 2 mya onwards) and up to 50-fold (assuming speech emergence in *H*. *sapiens*, 200 kya)^31^. Full articulatory range and excellent vocal control as observed today in humans may be relatively recent within the human lineage. However, the presence of learned consonant- and vowel-like calls, potentially as far as 10 mya within our lineage, allows considering gradual forces and progression in stages towards full-blown language. This intriguing possibility raises caution in the inference of the vocal capacities of extinct hominoidae from the fossil record without complementary assessment of the vocal capacities of extant great apes.

Vocal control over laryngeal and supralaryngeal structures at the root of a 10 mya timeline for spoken language evolution suggests that vocal evolution could have co-evolved with cognition within the human lineage. Whereas monkey cognitive skills have been hitherto assumed to surpass their vocal counterparts^32^,^33^, the possibility that the two skillsets originally exhibited even levels of sophistication in an ancestral hominid opens new considerations on speech/language evolution. In this scenario, vocal control would have allowed the immediate manifestation, or “verbalization,” of advanced cognition. Forces propelling cognitive processes would have then compelled vocal progress by association, and vice versa. For instance, with the emergence of theory of mind, individuals would have been able to exploit deceptive calls^34^,^35^,^36^, effectively launching new communicative and social dynamics within a population where acoustic deception was previously absent. If vocal and cognitive sophistication developed hand-in-hand over the course of human evolution during the last 10 mya, then, the processes of speech evolution and language evolution could be considered to have been one and the same. This “speech-language co-evolution” hypothesis will require future examination but it may perhaps expedite, for example, our understanding on the evolution of syntax and semantics. Because vocal control allows a functional divide between a signal (signifier^35^) and its functional use or meaning (signified^35^) – as suggested in our results – there would be few articulatory limitations for the assemblage of vocal sequences and the attribution of their respective informational content, so long as we had the required cognitive machinery to do so. In other words, in a condition where vocal evolution kept close pace with cognition, a human ancestor (regardless his/her position along human evolution timeline) would rarely have cognitive computations for which there were no matching vocal counterparts.

## Conclusion

We demonstrate real-time, dynamic and interactive vocal fold control beyond the vocal range of the orang-utan genus. This study offers a new category of vocal learning in great apes, in addition to previous cases describing gradual (over the course of months) and directional shift (exclusively downwards in frequency) of a species-specific vocalization^10^. Orang-utans (and possibly other great apes) possess a latent capacity for controlled deployment of vocal fold oscillation, allowing the volitional production of novel vowel-like calls. Theoretically, together with the capacity of great apes to socially learn voiceless consonant-like calls^3^,^20^, this proto-linguistic capacity constituted a crucial prerequisite for the onset of spoken language evolution.

## Additional Information

**How to cite this article**: Lameira, A. R. *et al*. Vocal fold control beyond the species-specific repertoire in an orang-utan. *Sci. Rep.* **6**, 30315; doi: 10.1038/srep30315 (2016).

## Supplementary Material

Supplementary Information

Supplementary Video

Supplementary Audio S1

Supplementary Audio S2
