# Supplementary material for: Vocal fold control beyond the species-specific repertoire in an orang-utan
Source: Sci Rep. 2016 Jul 27;6:30315. doi: 10.1038/srep30315 (PMC4962094; doi:10.1038/srep30315)
Supplement: Supplementary Information [file srep30315-s1.pdf]

Supplementary material

Vocal fold control beyond the species-specific repertoire in an orangutan

Adriano R. Lameira, Madeleine E. Hardus, Alexander Mielke, Serge A. Wich, Robert W. Shumaker

Supplementary table. 1. Descriptive statistics of orang-utan wookies and grumphs

	Call	N	Median	1st qrt	3rd qrt	min	max
Duration (s)	Wookie	124	0.381	0.252	0.515	0.134	1.24
	Grumph	39	0.164	0.144	0.2	0.059	0.287
Median pitch (Hz)	Wookie	124	117.4	103.8	131.8	68.6	180.3
	Grumph	39	219.9	120.1	248.3	75.7	292.2
Mean pitch (Hz)	Wookie	124	112.4	102.6	130.1	76.8	182.5
	Grumph	39	210.4	149.4	246.6	76.7	294.9
S.D. pitch (Hz)	Wookie	124	15.8	9.6	24.9	3.2	46.4
	Grumph	39	34.7	13.8	64.5	1.4	111.7
Min pitch (Hz)	Wookie	124	76.1	65.2	104.3	57.1	160.6
	Grumph	39	114.6	80.7	205.4	63.9	259.3
Max pitch (Hz)	Wookie	124	145.4	136.1	168	98.5	295
	Grumph	39	275.2	246.3	307.4	80.7	417.2
Pitch amplitude (Hz)	Wookie	124	63.2	42.4	91.1	11.3	223.5
	Grumph	39	117.5	53.9	198.8	3.4	267
F1 (Hz)	Wookie	124	481.1	408.9	544.8	218	620
	Grumph	39	302.7	266.5	367	250	578
F2 (Hz)	Wookie	124	1283.7	1138.7	1415.4	566	1708
	Grumph	39	1259.1	1199.8	1421.8	914	1515
F3 (Hz)	Wookie	124	1953.3	1761.3	2197.2	1180	2550
	Grumph	39	1991	1863.5	2106.2	1489	2260

Supplementary table 2. Descriptive statistics of orang-utan wookies sub-variants

	Sub-variant	N	Median	1st qrt	3rd qrt	min	max
Max. frequency (Hz)	Low	272	126	117.2	137.7	76.2	527.3
	Spontaneous	124	134.8	123	161.9	99.6	1418
	High	243	161.1	143.6	184.6	117.2	1183.6
Duration (s)	Low	272	0.252	0.203	0.317	0.11	0.8
	Spontaneous	124	0.381	0.252	0.515	0.13	1.24
	High	243	0.307	0.252	0.387	0.11	0.76
Max. power (dB)	Low	272	150.2	146.2	153.5	133.8	160.2
	Spontaneous	124	152.7	149.5	155.1	136	161.1
	High	243	156.9	155.2	158.4	148	163.7

Video/audio file legends

Movie S1 – Example of a match trial series between human demonstrator and Rocky.

Audio File S1 – Typical example of a high frequency wookie human-version followed by a match wookie by Rocky.

Audio File S2 – Typical example of a low frequency wookie human-version followed by a match wookie by Rocky.
